# The role of sperm-specific glyceraldehyde-3-phosphate dehydrogenase in the development of pathologies—from asthenozoospermia to carcinogenesis

**DOI:** 10.3389/fmolb.2023.1256963

**Published:** 2023-08-30

**Authors:** Irina Naletova, Elena Schmalhausen, Barbara Tomasello, Denis Pozdyshev, Francesco Attanasio, Vladimir Muronetz

**Affiliations:** ^1^ Institute of Crystallography, National Council of Research, Catania, Italy; ^2^ Belozersky Institute of Physico-Chemical Biology, Lomonosov Moscow State University, Moscow, Russia; ^3^ Department of Drug and Health Sciences, University of Catania, Catania, Italy; ^4^ Butlerov Chemical Institute, Kazan Federal University, Kazan, Russia

**Keywords:** GAPDS, GAPDH, sperm motility, melanoma, neurodegenerative diseases

## Abstract

The review considers various aspects of the influence of the glycolytic enzyme, sperm-specific glyceraldehyde-3-phosphate dehydrogenase (GAPDS) on the energy metabolism of spermatozoa and on the occurrence of several pathologies both in spermatozoa and in other cells. GAPDS is a unique enzyme normally found only in mammalian spermatozoa. GAPDS provides movement of the sperm flagellum through the ATP formation in glycolytic reactions. Oxidation of cysteine residues in GAPDS results in inactivation of the enzyme and decreases sperm motility. In particular, reduced sperm motility in diabetes can be associated with GAPDS oxidation by superoxide anion produced during glycation reactions. Mutations in GAPDS gene lead in the loss of motility, and in some cases, disrupts the formation of the structural elements of the sperm flagellum, in which the enzyme incorporates during spermiogenesis. GAPDS activation can be used to increase the spermatozoa fertility, and inhibitors of this enzyme are being tried as contraceptives. A truncated GAPDS lacking the N-terminal fragment of 72 amino acids that attaches the enzyme to the sperm flagellum was found in melanoma cell lines and then in specimens of melanoma and other tumors. Simultaneous production of the somatic form of GAPDH and sperm-specific GAPDS in cancer cells leads to a reorganization of their energy metabolism, which is accompanied by a change in the efficiency of metastasis of certain forms of cancer. Issues related to the use of GAPDS for the diagnosis of cancer, as well as the possibility of regulating the activity of this enzyme to prevent metastasis, are discussed.

## 1 Introduction: isoenzymes GAPDH and GAPDS: general information

D-Glyceraldehyde-3-phosphate dehydrogenase (EC 1.2.1.12) is a glycolytic enzyme catalyzing oxidative phosphorylation of glyceraldehyde-3-phosphate yielding 1, 3-diphosphoglycerate that is further used by phosphoglycerate kinase to produce ATP. Besides, glycolysis results in the production of pyruvate and NADH that are substrates for mitochondria.

Therefore, the enzyme is necessary for producing energy for the cell. In mammals, there are two isoenzymes: somatic enzyme (GAPDH) and sperm enzyme (GAPDS). Both GAPDH and GAPDS are homotetramers with the sequence identity of about 70%**.** They are encoded by different genes (*GAPD-1* and *GAPD-2*, respectively), which emerged after duplication of the original gene during the early evolution of chordates. The *GAPD-2* gene was then lost by most lineages, except for lizards, mammals, as well as cartilaginous and bony fishes. In lizards and mammals, GAPDS specialized to a testis-specific protein (Kuravsky et al., 2011)**.**

GAPDH is present in all tissues of the organism and localized in the cell cytoplasm. The importance of this enzyme is evidenced by its presence in the cells of all organisms, from prokaryotes to higher eukaryotes, and in very high concentrations. The content of GAPDH in the cytoplasm of cells is from 5% to 15% of the total amount of soluble proteins. The enzyme consists of 4 identical subunits of 36 kDa. Each subunit of human muscle GAPDH consists of 335 amino acid residues (UniProtKB/Swiss-Prot ID: G3P_HUMAN). The active site of the enzyme contains a highly reactive cysteine residue (Cys152) that is involved in catalysis. For a long time, it was considered that there is so much active GAPDH in cells that there is no need to regulate its activity and it always copes with its functions. However, the catalytic cysteine residue can be easily affected by different oxidants, resulting in the complete loss of the dehydrogenase activity (Little and O'Brien, 1969)**.**

GAPDS is a specific isoenzyme that is normally present only in sperm cells. It is detected in early spermatids and in mature spermatozoa of mammals and lizards (Feiden et al., 2008)**.** GAPDS of mammals possesses an additional N-terminal domain of 72–105 amino acid residues with a high content of hydrophobic residues (Bunch et al., 1998)**.** It was demonstrated that the N-terminal domain provides binding of GAPDS to the fibrous sheath, a cytoskeleton structure surrounding the axoneme in the principal piece of the sperm flagellum (Westhoff and Kamp, 1997; Bunch et al., 1998; Welch et al., 2000)**.** Investigation of recombinant GAPDS showed that the enzyme is inactivated in the presence of H_2_O_2_ with the same rate constant as rabbit muscle GAPDH (9 ± 0.3 and 10 ± 1.2 M^-1^ s^-1^ at pH 7.5, respectively (Elkina et al., 2010). Thus, both isoenzymes are sensitive to oxidation.

## 2 Energy source for sperm motility

Mammalian spermatozoa use carbohydrates (Mann and Lutwak-Mann, 1981). This makes it possible to produce ATP using both glycolysis and oxidative phosphorylation. In the vast majority of animal organisms, the energy for the movement of spermatozoa is provided by the isoenzyme GAPDH that is present in the somatic cells of the organism. The only exceptions are mammals and lizards, which have a special isoenzyme GAPDS in their spermatozoa.

The association with the fibrous sheath of the flagellum may indicate an important role of GAPDS in sperm motility. Several authors have proposed that the energy for the motility of mammalian spermatozoa is provided mainly by glycolysis. This assumption was based on experiments showing that the addition of mitochondrial inhibitor carbonyl cyanide m-chlorophenylhydrazine (CCCP) to highly active mice spermatozoa had no effect on ATP content and motility parameters (Mukai and Okuno, 2004). In addition, other research group showed that the deletion of GAPDS gene resulted in significant loss of ATP and blocking progressive motility of the sperm, although mitochondrial functioning was unchanged (Miki et al., 2004)**.** The above facts suggested that glycolysis is the preferred energy source for motility functions. However, later it was shown that the pathways of ATP production in spermatozoa (glycolysis or oxidative phosphorylation) can differ significantly in different mouse species, and the use of oxidative phosphorylation makes it possible to produce more ATP, which gives an advantage in the sperm velocity (Tourmente et al., 2015). The results of this study revealed the existence of significant variance in the bioenergetics metabolism. Presumably, pathways of energy production in human spermatozoa may be either glycolysis or mitochondrial respiration, or could take place in combination, depending on the surrounding environment and the availability of substrates (du Plessis et al., 2015).

## 3 Role of GAPDS in sperm motility

Since not only ATP is produced during glycolysis, but also pyruvate, which is a substrate for mitochondria, a lack of GAPDS can reduce ATP production resulting in a decrease in sperm motility. Investigation of the expression of GAPDS gene in 58 normokinetic and in 58 hypokinetic samples of human sperms shows that the expression profiles are similar in both groups (Paoli et al., 2017). This suggests that in most cases, asthenozoospermia is not associated with a low expression of the GAPDS gene (the exception is severe genetic abnormalities (Elkina et al., 2017). However, this does not rule out a possible effect of post-translational modifications of GAPDS on its activity. As mentioned in Section 1, GAPDS is oxidized in the presence of H_2_O_2_, which leads to inactivation of the enzyme (Elkina et al., 2010).

Based on these data, it was suggested that GAPDS may be oxidized *in vivo* under unfavorable conditions, and this oxidation may affect sperm motility. Two groups of semen samples (normokinetic and hypokinetic) were assayed for GAPDS activity. The mean value of GAPDS activity in hypokinetic sperm samples was found to be 2.5-3-fold lower than that in normokinetic samples. In addition, incubation of normokinetic sperm samples with H_2_O_2_ decreased sperm motility in dose dependent manner, which correlated with the decrease in GAPDS activity (*r* = 0.96) (Elkina et al., 2011).

These experiments show that GAPDS activity is important for sperm motility, and a decrease in the motility may be due to GAPDS oxidation. It is likely that a similar process can be observed *in vivo* under unfavorable conditions, which are accompanied by increased production of reactive oxygen species (ROS). In particular, increased ROS production is characteristic of diabetes. Diabetes leads to accumulation of methylglyoxal, a product of glucose metabolism, in the blood (Thornalley, 1996). Methylglyoxal interacts with the amino groups of proteins, resulting in the formation of cross-linked products. These products catalyze the formation of the superoxide anion, which oxidizes SH groups in proteins (Lee et al., 1998). Incubation of the somatic isoenzyme GAPDH with methylglyoxal results in an irreversible inactivation of the enzyme due to the oxidation of the catalytic cysteine residues yielding cysteine-sulfinic acid (Barinova et al., 2023). These data suggest a possibility of oxidation of GAPDS in diabetes by the same mechanism. Investigation of semen samples from patients with diabetes and healthy men showed that the mean values of GAPDS activity and sperm motility were lower in semen samples from diabetic patients than in normal samples. The values of the enzymatic activity of GAPDS showed significant positive correlation with sperm motility (*R* = 0.96) and significant negative correlation with the incidence of infertility (*R* = −0.99) (Liu et al., 2015). Taking into account the sensitivity of isoforms GAPDH and GAPDS to oxidation, the similarity of the structure of their active centers and the same catalytic mechanism, it can be assumed that increased production of ROS in diabetes results in oxidation of the catalytic cysteine residues in GAPDS, leading to inhibition of glycolysis and to a decrease in the sperm motility.

## 4 Expression of truncated GAPDS in cancer

As already mentioned in Section 1, full-length protein GAPDS is normally found only in mammalian spermatozoa, where it is tightly associated with the cytoskeleton of the sperm flagellum through the N-terminal domain. The exceptions are lizards, in which GAPDS is present not only in spermatozoa, but also in regenerating somatic tissues characterized by intensive cell division, and cancer cells in humans. However, in the case of expression in regenerating and cancer tissues, GAPDS loses its N-terminal domain, resulting in production of truncated GAPDS (t-GAPDS). It is well known that disruption of gene expression in oncological diseases leads to the production of proteins in cancer cells that are normally occurs only in spermatozoa. Such proteins constitute a large subgroup of cancer/testis antigens (Simpson et al., 2005). For example, the sperm-specific lactate dehydrogenase С gene has been shown to be expressed in late-stage breast cancer cells (Simpson et al., 2005), as well as in renal cell carcinoma samples (Hua et al., 2017). Moreover, the oncogenic role of this gene has been shown for these types of cancer. Another example is TPTE (Transmembrane Phosphatase with Tensin Homology), whose gene is predominantly expressed in spermatocytes and in various cancers, such as prostate cancer and hepatocellular carcinoma (Dong et al., 2003; Singh et al., 2008). The production of sperm-specific protein GAPDS in somatic tissues in some cancer diseases is another example of gene expression disorder. The presence of t-GAPDS in cancer cells was first shown in some melanoma lines (Sevostyanova et al., 2012). Analysis of the expression of GAPDS mRNA in different cancer cell lines showed a high content of GAPDS mRNA in some lines of melanoma cells (ArrayExpress database, www.ebi.ac.uk/array-express, accession numbers E-MTAB-37, E-MTAB-62, E-GEOD- 10843 and E-GEOD-7127). Subsequent analysis of three melanoma cell lines by Western blotting revealed t-GAPDS (without N-terminal fragment of 72 amino acid residues) in all samples. These results were confirmed by immunoprecipitation, mass spectrometry analysis, and immunochemical staining of melanoma cells with antibodies against GAPDS. Probably, as in the case of regenerating tissues in lizards (Kuravsky et al., 2011), t-GAPDS in melanoma loses its N-terminal fragment due to alternative splicing of the coding mRNA.

Interestingly, immunoprecipitation of proteins from melanoma cell extracts with the anti-GAPDS antibodies allowed isolation of a tetrameric protein containing both subunits of t-GAPDS and subunits of somatic GAPDH. It was suggested that the simultaneous production of two isoenzymes of glyceraldehyde-3-phosphate dehydrogenase not only stimulates glycolysis in melanoma cells, but also alters the induction of apoptosis, which involves only the somatic enzyme. Analysis of the relationship between the content of t-GAPDS and the intensity of glycolysis in 13 melanoma cell lines showed that both glucose consumption and lactate production were maximal in cells with the highest content of t-GAPDS. At the same time, a high content of t-GAPDS was characteristic of moderately differentiated melanoma cell lines. The influence of t-GAPDS production in cells on the regulation of the ratio of glycolysis, glutaminolysis and oxidative phosphorylation was also shown (Melnikova et al., 2020).

Subsequent studies showed that t-GAPDS are present not only in melanoma cell lines, but also in neoplasms themselves. Expression of t-GAPDS was revealed in uveal melanoma. Functional knockdown of GAPDS gene in uveal melanoma cell lines prevented cell growth and proliferation due to the slowing down glycolysis. Conversely, overexpression of t-GAPDS increased glucose uptake and production of lactate and ATP, leading to cell growth and proliferation. The authors concluded that t-GAPDS, which is regulated by the transcription factor SOX10, controls glycolysis and promotes tumorigenesis in uveal melanoma (Ding et al., 2021). In patients with stage III cutaneous melanoma, expression of GAPDS was shown to be associated with a poor prognosis (Falkenius et al., 2013). In another research, the authors tried to link the expression of t-GAPDS with the regulation of melanoma metastasis (Gill et al., 2022). Significant amounts of t-GAPDS were revealed in primary melanoma tumors. However, in metastasizing melanoma cells, the content of t-GAPDS and the intensity of glycolysis decreased with a simultaneous increase in the metabolites of the tricarboxylic acid cycle. Overexpression of t-GAPDS blocked metastasis, and its inhibition promoted metastasis, reduced glycolysis, and increased levels of several metabolites of the tricarboxylic acid cycle and their derivatives, including citrate, fumarate, malate, and aspartate.

To understand the role of t-GAPDS in the metabolism of cancer cells, it is important to study its catalytic properties using a recombinant form of the protein. To obtain soluble and active recombinant GAPDS, it was necessary to remove its N-terminal domain, which serves to attach GAPDS to the cytoskeleton of the sperm flagellum, since when expressed in *E. coli* cells, the hydrophobic N-terminal domain prevents the normal folding of the protein and promotes its aggregation. Thus, a recombinant GAPDS protein without the N-terminal domain (dN-GAPDS) was obtained and investigated (Elkina et al., 2010). It should be noted that the recombinant dN-GAPDS is similar to t-GAPDS found in cancer tumors. The recombinant protein dN-GAPDS is enzymatically active, and its catalytic parameters do not differ much from those of somatic GAPDH. An unusual property of dN-GAPDS is an increased stability compared to somatic isoform GAPDH (Elkina et al., 2010; Kuravsky et al., 2014)**.** Consequently, the influence of t-GAPDS on the intensity of glycolysis and other cell vital processes may be associated with its high stability, which may increase the lifetime of the protein and ensure a greater total activity of this enzyme compared to GAPDH.

Thus, we can make an unambiguous conclusion that the production of the t-GAPDS plays an important role in providing energy to growing melanoma cells due to the intensification of the glycolytic pathway. However, melanoma metastasis is associated with low t-GAPDS activity accompanied by an increase in the TCA cycle. Consequently, the use of GAPDS inhibitors to suppress the growth of melanoma as previously proposed (Ding et al., 2021) may lead to undesirable consequences. Inhibition of t-GAPDS may increase metastasis, which is even more dangerous than an increase in the size of tumors. Before using t-GAPDS as a target for the treatment of melanomas, a more thorough study of all aspects of the participation of this enzyme in the metabolism of cancer cells is necessary. However, already at this stage, t-GAPDS can be used as a marker protein not only for diagnosing melanomas, but also for determining the stage of development of this disease.

It should also be noted that data on the presence of GAPDS mRNA in lung cancers have recently been published. The GAPDS gene was among 24 genes related to mitochondrial energy metabolism, with significantly increased transcription in these cancers (Ye et al., 2021). In addition, according to immunohistochemistry data from the Human Protein Atlas project, a few cases of testicular cancer and a single case of prostate cancer showed weak to moderate nuclear staining with anti-GAPDS antibodies (Uhlen et al., 2017).

To summarize the above, full-length GAPDS is found only in sperm cells. Reports on GAPDS detection in somatic tissues describe GAPDS lacking the N-terminal domain (truncated GAPDS, t-GAPDS). The N-terminal domain is required for GAPDS binding to the cytoskeleton of the sperm flagellum, and its removal has no effect on GAPDS enzymatic activity, but allows production of GAPDS in a soluble form. Truncated GAPDS is found in regenerating tissues of lizards and in cancer cells in humans. We would like to note the relationship between the production of t-GAPDS and intensive cell division. While in lizards the appearance of t-GAPDS leads to positive effects by enabling tissue regeneration, in mammals, at least in the case of melanoma, t-GAPDS production stimulates the development of malignant tumors. It is possible that the enhanced stability of t-GAPDS compared to GAPDH gives regenerating and cancer cells an advantage in energy production via glycolysis.

## 5 GAPDS and neurodegenerative diseases

It cannot be ruled out that not only cancerous, but also other transformations of somatic cells can lead to the appearance of GAPDS. So, meta-analysis of Alzheimer disease (AD) genetic association studies has shown that among three hundred genes that are presumably associated with Alzheimer’s disease only about 10 are sufficiently reliable participants in the development of this pathology (Bertram et al., 2007). One of these genes is responsible for the production of GAPDS. The study of single nucleotide polymorphisms among GAPDH paralogs including the GAPDS gene revealed their association with the risks of developing Alzheimer’s disease. In addition, transcription of the GAPDS gene was detected in brain tissues (Li et al., 2004). Unfortunately, there is currently no experimental confirmation of the participation of GAPDS in the occurrence and development of neurodegenerative diseases. However, direct binding of non-native somatic GAPDH with amyloid-beta peptide (Aβ) was shown (Naletova et al., 2008), as well as stimulation of aggregation of such peptides in the presence of GAPDH aggregates (Itakura et al., 2015). Moreover, aggregates containing GAPDH and Aβ have been found in the cerebrospinal fluid of Alzheimer’s patients (Lazarev et al., 2021). Thus, modification of GAPDH by reactive oxygen/nitrogen species affects not only energy metabolism, but also leads to stimulation of amyloidogenesis.

The relationship between the development of neurodegenerative diseases, oxidative stress, and the role of copper ions indicates that GAPDH can be a trigger for this process due to the presence of the sulfhydryl group sensitive to various modifications in this enzyme. It is widely recognized that copper ions not only interact and form complexes with Aβ but are also involved in the aggregation of amyloidogenic peptides (Thakur et al., 2011; Attanasio et al., 2013b). Many peptide inhibitors of Aβ aggregation (Attanasio et al., 2013a; Wang et al., 2014; Barrera Guisasola et al., 2015; Greco et al., 2020) and copper chelators (Tabbì et al., 2015; Magrì et al., 2016; Sciacca et al., 2022) have been proposed for the treatment of AD (Ryan et al., 2018). Moreover, a significant inhibitory effect on GAPDH activity was obtained with copper that suggests the involvement of mainly catalytic cysteine and neighboring histidine residues in the binding of this metal (Krotkiewska and Banaś, 1992).

Whether it is the somatic GAPDH or GAPDS that is involved in these processes has not been specially studied, but the meta-analysis data described above indicate a possible involvement of GAPDS.

## 6 Conclusion and perspectives

**FIGURE 1:**
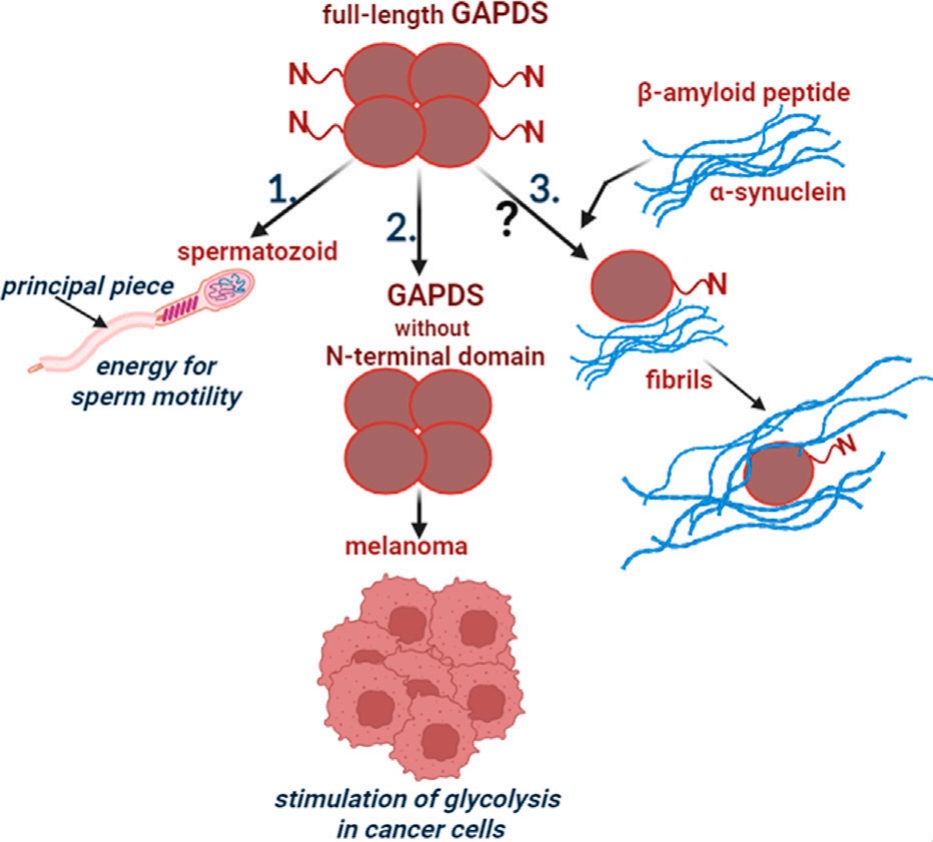
Participation of GAPDS in normal and pathological processes. 1. The full-length GAPDS is necessary for sperm motility: the N-terminal domain provides attachment of GAPDS to the cytoskeletal structure in the principal piece of the sperm flagellum. 2. The truncated GAPDS without the N-terminal domain stimulates glycolysis in melanoma. 3. Few data suggest participation of GAPDS in the development of neurodegenerative diseases, but the mechanism is unclear.

Thus, GAPDS not only performs its glycolytic function when supplying energy to mammalian spermatozoa, but also participates in the regulation of glycolysis in cancer cells. It is also possible that GAPDS is directly involved in the formation of amyloid structures that are characteristic of neurodegenerative diseases (Figure 1).

## Abbreviations

Aβ, amyloid-beta peptide; AD, Alzheimer disease; ATP, adenosine triphosphate; GAPDH, glyceraldehyde-3-phosphate dehydrogenase, somatic isoenzyme; GAPDS, glyceraldehyde-3-phosphate dehydrogenase, sperm-specific isoenzyme; t-GAPDS, truncated form of GAPDS.
